# Utilizing Project ECHO-Geriatrics for Interprofessional Education of Health Professions Students and Trainees

**DOI:** 10.1093/geroni/igaf122.750

**Published:** 2025-12-31

**Authors:** Katherine Bennett, Ashley McPeek, Noa Brazg

**Affiliations:** University of Washington School of Medicine, Seattle, Washington, United States; University of Washington Center for Health Sciences Interprofessional Education, Research, and Practice, Seattle, Washington, United States; University of Washington, Seattle, Washington, United States

## Abstract

With the rapid expansion of the Project ECHO model over the past decade, there have been increasing numbers of ECHO programs that include interprofessional expert panels. Observation of interprofessional clinical practice is not readily available to most health professions students, and ECHO provides a venue for observing and participating in discussions that exemplify interprofessional teamwork. The University of Washington’s Project ECHO-Geriatrics has run monthly from 2016 to the present to increase geriatrics education for family medicine residents throughout a regional residency network. It includes a robust panel of health professionals, including geriatricians, psychiatrists, nurses, pharmacists, social workers, and Area Agency on Aging staff members. Each session includes a brief didactic, followed by an interprofessional case discussion of a patient presented by a program participant. In 2022, a program was launched for UW health professions trainees to participate longitudinally in Project ECHO-Geriatrics. Students attend an introduction to Project ECHO and Age-Friendly Care and then participate in at least three monthly Project ECHO-Geriatrics Sessions that are followed by a separate debrief for students. Health professions students across ten disciplines (e.g., medicine, nursing, pharmacy, social work, dentistry, and public health) have participated thus far. Surveys of participants have consistently shown increased confidence in both interprofessional teamwork and geriatrics skills as a result of program participation. These programs at UW for trainees and students offer a framework for the growing number of ECHOs with interprofessional panels to leverage their existing program to increase geriatrics and interprofessional team skills among trainees across many health professions.

